# Bat Airway Epithelial Cells: A Novel Tool for the Study of Zoonotic Viruses

**DOI:** 10.1371/journal.pone.0084679

**Published:** 2014-01-13

**Authors:** Isabella Eckerle, Lukas Ehlen, René Kallies, Robert Wollny, Victor M. Corman, Veronika M. Cottontail, Marco Tschapka, Samuel Oppong, Christian Drosten, Marcel A. Müller

**Affiliations:** 1 Institute of Virology, University of Bonn Medical Centre, Bonn, Germany; 2 Institute of Experimental Ecology, University of Ulm, Ulm, Germany; 3 Smithsonian Tropical Research Institute, Balboa, Panama; 4 Kwame Nkrumah University of Science and Technology, Kumasi, Ghana; NIH, United States of America

## Abstract

Bats have been increasingly recognized as reservoir of important zoonotic viruses. However, until now many attempts to isolate bat-borne viruses in cell culture have been unsuccessful. Further, experimental studies on reservoir host species have been limited by the difficulty of rearing these species. The epithelium of the respiratory tract plays a central role during airborne transmission, as it is the first tissue encountered by viral particles. Although several cell lines from bats were established recently, no well-characterized, selectively cultured airway epithelial cells were available so far. Here, primary cells and immortalized cell lines from bats of the two important suborders Yangochiroptera and Yinpterochiroptera, *Carollia perspicillata* (Seba's short-tailed bat) and *Eidolon helvum* (Straw-colored fruit bat), were successfully cultured under standardized conditions from both fresh and frozen organ specimens by cell outgrowth of organ explants and by the use of serum-free primary cell culture medium. Cells were immortalized to generate permanent cell lines. Cells were characterized for their epithelial properties such as expression of cytokeratin and tight junctions proteins and permissiveness for viral infection with Rift-Valley fever virus and vesicular stomatitis virus Indiana. These cells can serve as suitable models for the study of bat-borne viruses and complement cell culture models for virus infection in human airway epithelial cells.

## Introduction

Emerging infectious diseases are perceived as a significant threat to human health. Among emerging infectious diseases, approximately 2/3 are of zoonotic origin [1]. While bats fulfill many ecologically important functions, several species were recognized as potential reservoirs of zoonotic viruses. Emerging human diseases caused by bat-borne viruses include the severe acute respiratory syndrome, Hendra and Nipah encephalitides, as well as Ebola- and Marburg hemorrhagic fever [2]–[4]. A recent outbreak of severe respiratory infection in the Middle East region caused by the novel human coronavirus MERS-CoV with close identity to bat coronaviruses emphasizes the relevance of bat-borne zoonotic diseases [5]–[7]. The conditions and mechanisms behind animal-to-human spillover are unknown.

Research on bats has led to the discovery of a plethora of novel bat virus sequences, including viruses that are presumably candidates for switching their host species due to a close identity with human viruses [8]–[11]. To assess the risk and potential for human pathogenicity of novel bat viruses, it is crucial to establish valid research tools for comparative *in vitro* infection modeling. The two main obstacles for studying novel bat viruses are: 1) isolation attempts by conventional methods in cell culture or animal models have been unsuccessful for many viruses; and 2) suitable model systems to study virus isolates in their natural host are missing. In most cases it is not possible to study virus replication in bats under experimental conditions, because species of interest cannot be kept or bred in captivity. Also, due to conservation reasons and ethical concerns, it is not justifiable to sample an arbitrary number of these animals from their natural habitat for infection studies in the laboratory.

To isolate and study zoonotic viruses with airborne transmission, cell culture models representing the respiratory tract are of special interest. During infection, the airway epithelium plays a crucial role: i) By forming a physical barrier between a host and its environment, these are often the first cells that encounter pathogens ii) they represent a barrier that must be crossed by the virus for successful entry into the host. The airway epithelium is not only a pure “physical” barrier consisting of a tightly packed layer of cells covered with mucus. Recent studies also show that airway epithelial cells provide a complex contribution to host defense, innate immunity, and immune regulation (for a review see [12]).

So far, only one cell line from the respiratory tract of a bat is widely available. This cell line is derived from the lung of *Tadarida brasiliensis* (Tb 1 Lu, American Type Culture Collection, ATCC Nr. CCL-88), but no information on the cell type of origin exists. We and others have recently established bat cell lines from a larger number of bat species and different organ types, which have already provided important insight into immunology and virus-host interaction of different zoonotic viruses [13]–[15]. To specifically address the role of the airway epithelium in virus-host interaction in the reservoir host, we present a further specification of bat cell culture models.

We chose two bat species which reflect the two bat suborders Yangochiroptera and Yinpterochiroptera, that both include species presenting properties relevant for zoonotic transmission: broad distribution range, high population densities, and in some species frequent interactions with humans due to a high adaptability to environments altered by humans. Seba's short-tailed fruit bat, *Carollia perspicillata*, a member of the highly diverse family Phyllostomidae (the largest and most diverse bat family in the Neotropics), is stable in its population trend, according to the International Union for the Conservation of Nature and Natural Resources (IUCN, www.iucnredlist.org). It is highly abundant in many localities of the Neotropics. The straw-colored fruit bat, *Eidolon helvum*, a member of the *Pteropodidae*, is found in very large colonies across a wide area in both East- and West Africa, and lives close to human settlements and in urban areas. In contrast to *C. perspicillata*, the population trend of *E. helvum* is suspected to be decreasing due to intensive hunting for meat (IUCN: near threatened), but the sometimes very large colonies and the adaptability to urban habitats pose potential risks for zoonotic transmission. Furthermore, *E. helvum* is capable of travelling thousands of kilometers across Africa within a seasonal migration pattern [16].

Of note, these characteristics are not limited to the two bat species named above but are true for many bat species around the world. Among them are typical characteristics of bats (i.e. large population size) that distinguish bats from other mammals as reservoirs for potentially zoonotic viruses.

To generate a tool for isolation of bat-borne viruses and to facilitate studies on virus transmission in the natural reservoir host, we aimed at establishing airway epithelial cell lines derived from bats.

## Methods

### Ethics statement

Capturing and sampling of *E. helvum* was done with permission from the Wildlife Division, Forestry Commission, Accra, Ghana. Geographic co-ordinates of the sampling site in Kumasi/Ghana were N06°42′02.0″ W001°37′29.9″. Under the auspices of Ghana authorities bats were caught with mist nets, anaesthetized with a Ketamine/Xylazine mixture and euthanized by cervical dislocation (permit no. CHRPE49/09; A04957) as described previously [14]. Veterinary skilled staff performed all procedures on *E. helvum*. Additional export permission was obtained from the Veterinary Services of the Ghana Ministry of Food and Agriculture (permit no. CHRPE49/09; A04957). For the generation of Yangochiroptera cell lines, the Institute of Zoology, University of Veterinary Medicine Hannover, Germany, kindly provided a post-mortem trachea sample of *C. perspicillata* from a breeding colony established for research purposes on Neurophysiology and Neurobiology of bats (Landeshauptstadt Hannover, Fachbereich Recht und Ordnung, Gewerbe und Veterinärangelegenheiten, permit no. 42500/1H). Animals were euthanized (permit No. 11/0435) by cervical dislocation while under halothane anesthesia.

### Cell culture

After the animals were euthanized as described above, tracheas were removed *in toto*. Trachea specimens were immediately placed in ice-cold medium for transport or, if longer transport was necessary (as in the case of samples of *E. helvum* from Ghana), frozen at −80°C in cell culture freezing medium (PAA, Pasching, Austria). The following steps were done under sterile conditions using a laminar flow hood. Upon arrival at the laboratory, frozen specimens were thawed and washed in 37°C warm, sterile phosphate buffered saline (PBS). The trachea and large bronchi were roughly cleaned from the attached surrounding tissue, and then longitudinally chopped into pieces with a sterile blade. Tissue fragments were placed in a 6-well cell culture plate and submerged in 37°C warm medium. The medium consisted of primary airway epithelial cell medium basal mix supplemented with bovine pituitary extract 0.004 ml/ml, epidermal growth factor (recombinant human) 10 ng/ml, insulin (recombinant human) 5 µg/ml, hydrocortisone 0.5 µg/ml, epinephrine 0.5 µg/ml, triiodo-L-thyronine 6.7 ng/ml, holo-transferrin (human) 10 µg/ml, and retinoic acid 0.1 ng/ml (Promocell, Heidelberg, Germany). The primary cell medium was additionally supplemented with penicillin/streptomycin (Life Technologies GmbH, Darmstadt, Germany), ofloxacin (Tarivid®, Sanofi-Aventis), and amphotericin B (PAA, Pasching, Austria) for the initial culturing of the tissue to avoid bacterial and fungal contamination. Any movement of the cell culture plates was avoided during the first 3 days, then cells were observed daily for quality of beating of ciliated cells and outgrowth of primary cells. After an outgrowth of primary cells was observed, the medium was changed every 2 days. When nearly confluent, cells were immortalized by lentiviral transduction of the large T antigen of SV40 as described previously [14], [15]. All cell cultures were genotyped by amplification of mitochondrial cytochrome c oxidase I [17].

After immortalization, cells were passaged frequently, at least once a week, until a sudden increase in cell growth was observed, at which point they were partly stock frozen for further use. After three to four passages, cells were characterized for expression of epithelial marker proteins cytokeratin and zonula occludens protein 1 (ZO-1) by immunofluorescence staining. Cells were subcloned by end-point-limiting dilution and those cell populations that were initially derived from a single cell and presented an epithelial morphology most closely resembling the primary cell morphology were selected. Characterization was repeated for all subclones. After subcloning, cells were adapted to a supplemented Dulbecco's modified Eagle's medium (DMEM) (PAA, Cölbe, Germany) with 10% heat-inactivated FBS as described previously [14]. The cell line Tb 1 Lu (American Type Culture Collection, ATCC Nr. CCL-88) was cultured under the same conditions. Cells were incubated at 37°C and 5% CO_2_.

### Nucleic acid preparation and real-time PCR

Viral RNA was extracted from the cell culture supernatant according to the manufacturer's instructions (Nucleospin® RNA virus, Machery Nagel, Düren, Germany). PCR was performed using the SuperScript® III One-Step RT-PCR System with Platinum® Taq DNA Polymerase (Invitrogen, Karlsruhe, Germany). Cycling conditions for the vesicular stomatitis virus Indiana (VSV) and Rift Valley fever virus (RVFV) real-time RT-PCR included a reverse transcription step for 15 min at 55°C, initial denaturation for 2 min at 95°C, and 45 cycles consisting of denaturation for 15 seconds at 95°C and primer annealing/elongation for 30 seconds at 58°C. Real-time RT-PCR was carried out using the LightCycler 480 Real-Time PCR System (Roche, Basel, Switzerland).

### Immunofluorescence assay (IFA)

Cells were seeded on glass slides and washed with PBS on the following day, then fixed with acetone-methanol. Slides were incubated with primary mouse monoclonal antibodies against pan-cytokeratin (mouse-monoclonal, Abcam, C-11, ab7753) and rabbit polyclonal antibodies against zonula occludens protein (ZO-1 Mid) (rabbit polyclonal, Invitrogen 40-2200) diluted 1∶400 in PBS overnight at 4°C. The secondary antibodies were cyanine 3-labeled donkey-anti-mouse serum and cyanine 2-labeled donkey-anti-rabbit serum (Dianova). Nuclei were counterstained with DAPI. All photographs were taken with a 207 Motic Axiovision microscope (Zeiss). Antibodies with reactivity to a broad variety of species were chosen. To compare bat and rodent staining to the staining in cells with confirmed reactivity, all antibodies were additionally tested in porcine airway epithelial cells. A comparable staining pattern was seen in all cells (data not shown). Cells were then subcloned by end-point-limiting dilution and multiple clones were selected for generation of homogeneous cell lines. After selection of clones, characterization by IFA was repeated in the subclones selected.

### Species confirmation

Although the species from which cells were obtained were identified by experienced field workers (*E. helvum*) or from an established breeding colony (*C. perspicillata*), species confirmation of the cell lines was performed by sequencing subunit I of the cytochrome c oxidase gene (CO1) and compared to available databases (GenBank and BOLD System, www.barcodinglife.com).

### Pathogen screening

As the cells were derived from feral animals or from breeding populations without special pathogen free (SPF) housing conditions, the immortalized cells were investigated for putative contaminants which might be cultured along with the cells, posing a risk for laboratory workers as well as a source of bias when assessing virus-host interaction. Cells were detached and dissociated with Accutase, and washed twice in PBS. Then, viral DNA/RNA was extracted as described above. Cells were tested for viral nucleic acids using generic (RT)-PCR assays specific for different virus families and genera. (RT)-PCR amplification products were pooled and analyzed by agarose gel electrophoresis. Amplicons ranging in size from 150 bp to 700 bp were cut from the gel and purified using the QIAEX II Gel Extraction Kit (Qiagen, Hilden, Germany). Fragment ends were repaired, 454 sequencing adaptors were ligated, and emulsion PCR was performed according to standard 454 sequencing protocols (Roche, Mannheim, Germany). Deep sequencing of (RT)-PCR products was done on a 454 Genome Sequencer Junior (Roche). The resulting reads were aligned against the virus database using the BLASTn (wordsize 7) and tBLASTx (wordsize 2) algorithms [18] with an e-value cut-off of 10^−3^. All cell lines were controlled for mycoplasma [19] and SV 5 (in-house assay, see also [14]). Additionally, cell lines were screened for lyssaviruses [20].

### Virus infection studies

Cells were seeded in 24-well plates at a density of 4×10^5^ cells per ml. The following day, cells were infected with VSV Indiana or RVFV clone 13 at a multiplicity of infection (MOI) of 0.1 or 0.001 as described previously [14], [21]. Briefly, the medium was removed and cells were infected with virus diluted in Optipro serum-free medium (Life Technologies GmBH, Darmstadt, Germany) for 1 h. Cells were inoculated for 1 h at 37°C and washed twice with PBS after infection. Samples were taken at time points 0, 4, 8, 12, 24, and 48 hours post infection (hpi) for VSV and 0, 8, 12, 24, 48, and 72 hpi for RVFV.

## Results

**Figure 1:**
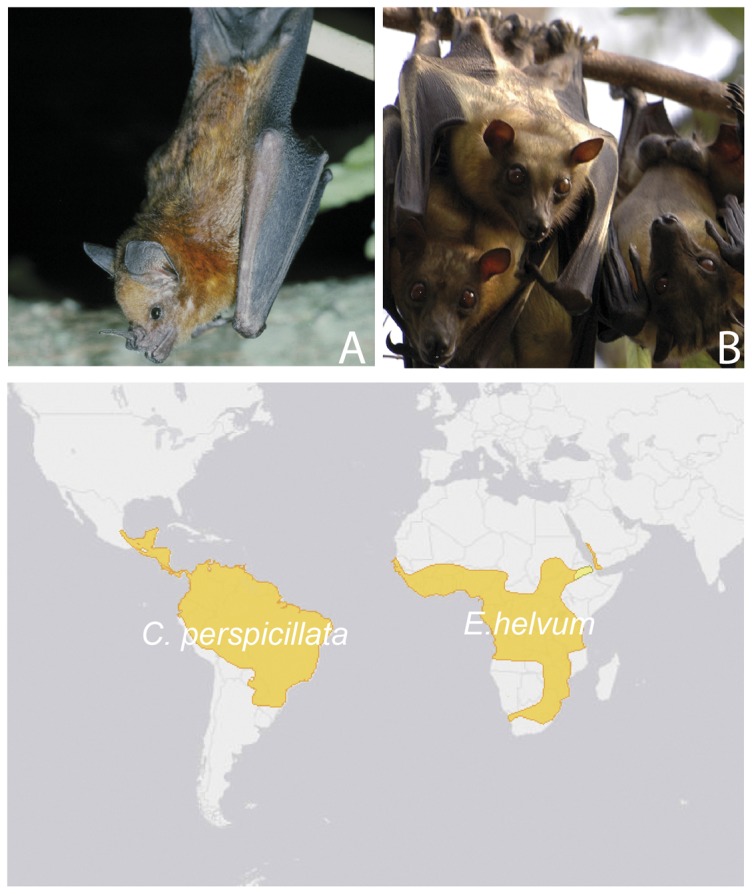
Species for which airway epithelial cells were established and their geographic distribution. A. Seba's short-tailed bat (*C. perspicillata*), and B. Straw-colored fruit bat (*E. helvum*) (upper row), and their distribution (lower row) (map adapted from IUCN Red List of Threatened Species, http://www.iucnredlist.org).

A protocol for isolation of primary airway epithelial cells was established and immortalized cell lines were generated from the two bat species, *C. perspicillata* and *E. helvum*, of the suborders Yangochiroptera and Yinpterochiroptera (**Table 1** **,** **Figure 1**). An extensive review of the literature identified a high diversity of viruses in both species, among them zoonotic viruses or viruses related to zoonotic viruses (**Table 2**).

**Table 1:** Characteristics of bat species chosen for the establishment of bat airway epithelial cells.

	Seba's short-tailed bat (*C. perspicillata*)	Straw-colored fruit bat (*E. helvum*)
Suborder	Yangochiroptera	Yinpterogochiroptera
Family	Phyllostomidae	Pteropodidae
Distribution	Middle- and South America	Africa, Arabian Peninsula
Habitat	Understory resident of rain forests and secondary forests, roosting in caves and hollow trees. Widespread and highly abundant in many localities of the Neotropics.	Tropical rain forest, dry savanna, modified habitats and urban areas. Animals may move during their seasonal migrations over thousands of km.
Diet	Predominantly frugivorous	Frugivorous
Migratory	no	yes
Colony size	10–100	100,000–1,000,000
Population trend	stable	decreasing
Status	Least concerned	Near threatened

**Table 2:** Viruses associated with the bat species *C. perspicillata* and *E. helvum*.

Bat species	Virus family	Virus genus	Virus identified	Reference
Seba's short-tailed bat *(C. perspicillata)*	Retroviridae	unassigned	unassigned	[33]
	Bunyaviridae	Orthobunyavirus	Bimiti virus	[34]
			Catu virus	[34]
			Marituba virus	[34]
	Flaviviridae	Flavivirus	Ilheus virus*	[34], [35]
			Tamana bat virus	[36]
			Dengue virus*	[36], [37]
			Rio Bravo virus*	[36]
			St. Louis encephalitis* virus	[34]
			Yellow fever virus*	[34]
	Togaviridae	Alphavirus	Eastern equine encephalitis virus*	[36]
			Venezuelan equine encephalitis virus*	[35], [38]–[40]
	Rhabdoviridae	Lyssavirus	Rabies virus*	[41]
		Vesiculovirus	Vesicular stomatitis Indiana virus*	[42]
			Vesicular stomatitis New Jersey virus*	[42]
	Paramyxoviridae	Morbillivirus-related	unassigned	[8]
	Paramyxoviridae	Morbillivirus-related	unassigned	[8]
	Polyomaviridae	Orthopolyomavirus	Bat polyomavirus	[43]
	Coronaviridae	Coronavirus	Bat Coronavirus	[44]–[46]
Straw-colored fruit bat (*E. helvum)*	Herpesviridae	unassigned	unassigned	[47]
		Simplexvirus	Eidolon helvum simplexvirus 1	[48]
		unassigned	several, unassigned	[49]
	Adenoviridae	unassigned	unassigned	[49]
	Reoviridae	Orbivirus	Ife virus	[38], [50]
		Rotavirus	Bat/KE4852/07	[51]
	Filoviridae	Ebolavirus	Ebola-Zaire virus*	[52]
	Paramyxoviridae	Henipavirus	Hendra virus*	[53]
		Henipavirus-like	Several, unassigned	[8], [54], [55]
		Rubulavirus	Achimota virus 1 and 2	[56]
			Several, unassigned	[8]
		Pneumovirus	Several, unassigned	[8]
	Papillomaviridae	unassigned	Several, unassigned	[49]
	Polyomaviridae	unassigned	Several, unassigned	[49]
	Picornaviridae	unassigned	Several, unassigned	[49]
	Poxviridae	unassigned	Several, unassigned	[49]
	Parvoviridae	Parv-4-like viruses	Eidolon-helvum-bat-Parvovirus 1	[57]
		unassigned	unassigned	[49]
	Retroviridae	unassigned	unassigned	[49]
	Rhabdoviridae	Lyssavirus	Lagos bat virus	[38]
			Mokola virus*	[58]

Viruses were detected either as virus isolates, on the basis of genetic sequence, or indirectly by detection of antibodies. Table adapted from [32]; modified and supplemented. Viruses that have been reported to infect humans are marked with *.

### Establishment of primary cells from outgrowths

**Figure 2:**
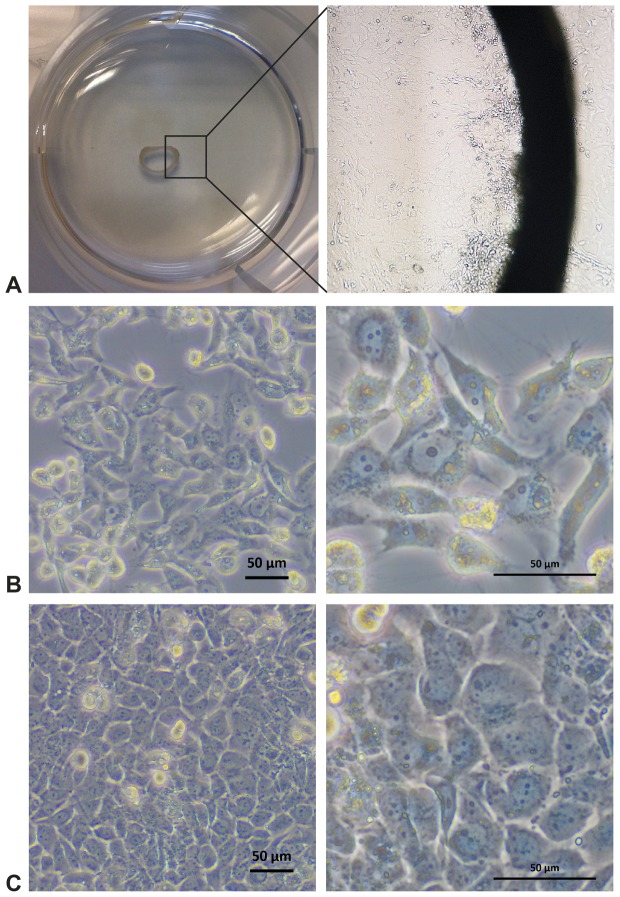
Establishment of airway epithelial cell culture by outgrowth from trachea specimens. **A** Trachea tissue sample in cell culture dish viewed from above (left side) with outgrowth of primary airway epithelial cells from the mucosal layer (right side, 100× magnification) (here: *E. helvum*) **B** Immortalized and subcloned airway epithelial cells from *E. helvum*, subclone 1 (designated EidheAEC.B-1) **C** Immortalized and subcloned airway epithelial cells from *C. perspicillata*, subclone 3 (designated CarperAEC.B-3). Black bars represent 50 µm.

Growth of primary cells from the tissue was observed between 3–5 days after tissue fragments were placed in the dish (see **Figure 2** **A, B**). Directly after placing the tissue fragments in the dish, beating of ciliated cells was observed on the luminal side of the tracheal rings from both fresh and frozen trachea specimens (see **Video S1**). Outgrowing cells displayed a homogeneous, cobblestone-like morphology typical of epithelial cells (see **Figure 2** **C**). To increase the number of primary cells, tracheal rings were carefully removed once the dish was fully coated with cells (usually after 2–3 weeks) and placed in a new dish, where repeated outgrowth of cells was observed. This procedure could be repeated five to six times; however, a decrease in the number of outgrowing cells as well as loss of active ciliated cells within the tracheal ring over time was observed. In general, outgrowth of cells from the tissue specimens was observed for more than 3 months when frequently placed in new dishes and supplemented with fresh medium every 2 days.

### Immortalization and epithelial characterization

**Figure 3:**
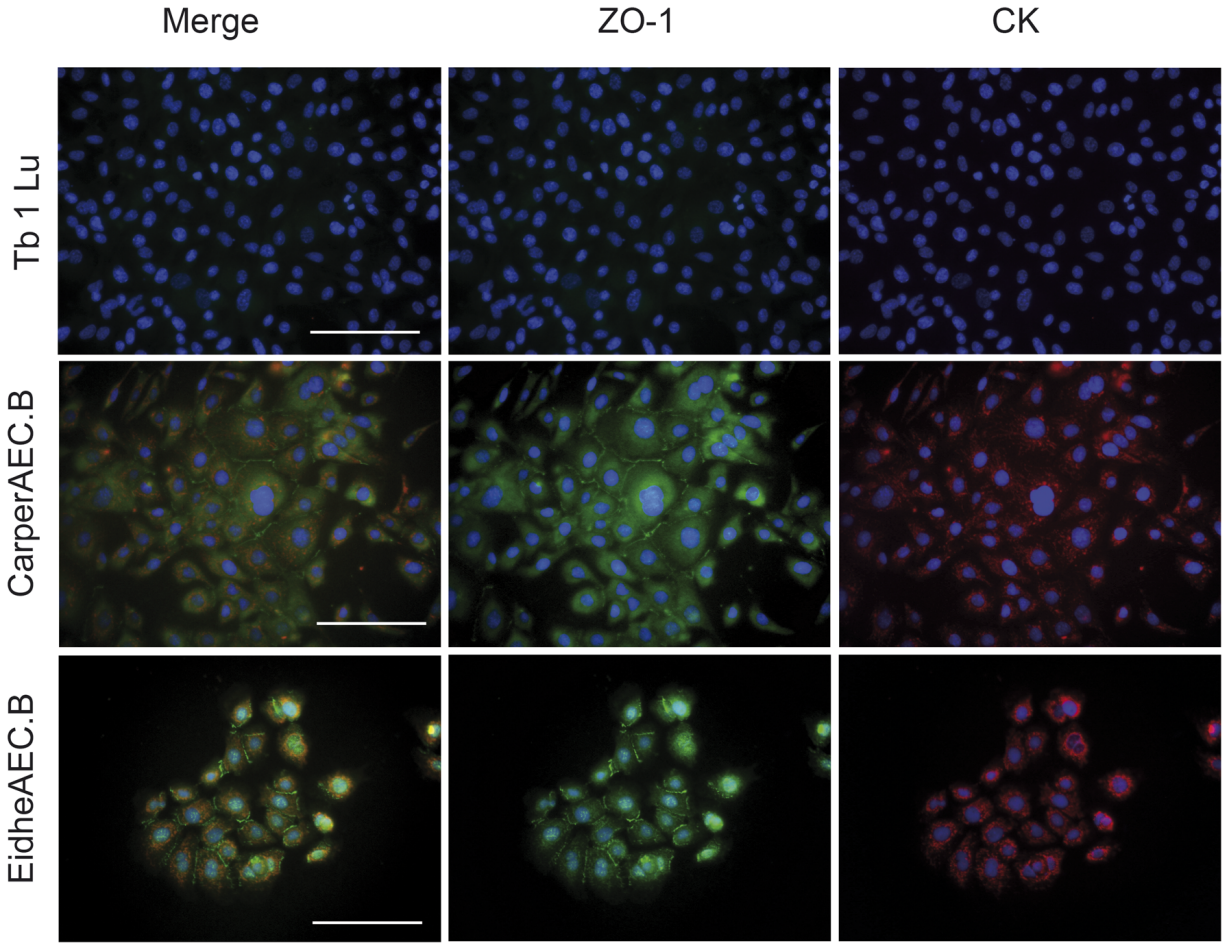
Immunofluorescence staining for markers of epithelial origin of Tb 1 Lu and airway epithelial cells from *C. perspicillata* (CarperAEC.B) and *E. helvum* (EidheAEC.B) prior to subcloning. The markers used to confirm epithelial origin were cytokeratin (CK, red) and zonula occludens-1 (ZO-1, green); nuclei are counterstained with DAPI (blue). Expression of both markers is present in all cell lines generated by the described methods, indicating an epithelial origin. By contrast, the commercially available Tb 1 Lu does not show expression of the respective markers.

Primary cells of both species were successfully immortalized by lentiviral transduction of the large T antigen of SV40. After immortalization, a decline in the cell population was observed. One to two weeks afterwards, surviving cells started to proliferate rapidly. Cells were expanded and characterized for epithelial cell markers by IFA with broadly reactive antibodies against cytokeratins (anti-pan-cytokeratin) as well as zonula occludens protein (ZO-1). The cells in both of the cell lines generated were positive for both of these epithelial markers, in contrast to the commercially available cell line Tb 1 Lu (see **Figure 3**).

### Species confirmation

The species of origin for both cell lines were confirmed by their CO1 sequences in the BOLD system.

### Screening of cells for viral contamination

Apart from testing all cells for lyssaviruses, 454 sequencing was used for broad pathogen screening. After lysis of cell pellets and purification of RNA and DNA, nucleic acid amplification was performed using degenerate primers targeting conserved regions of major virus families or genera (**Table S1**). All sequences obtained were identified as host sequences; no sequences from viral or bacterial organisms were found.

### Virus infection studies

**Figure 4:**
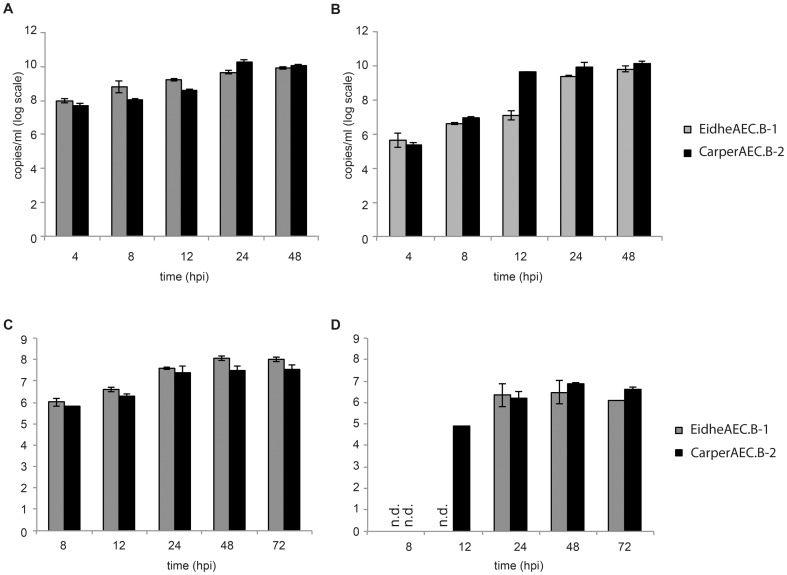
Virus infection studies with vesicular stomatitis virus (VSV) (A, B) and Rift Valley fever virus (RVFV) (C, D), and at MOI of 0.1 (left side) and 0.001 (right side), in subclones of immortalized *C. perspicillata* airway epithelial cells (subclone 3, designated CarperAEC.B-3) and *E. helvum* airway epithelial cells (subclone 1, designated EidheAEC.B-1). Viruses were detected by quantitative real-time RT-PCR. All cells were infectable and able to support replication of RVFV and VSV.

To investigate whether the cells generated were permissive for virus infection, we performed virus infection studies with two zoonotic viruses, VSV and RVFV clone 13 **(** **Figure 4** **)**. Cells were infected with two different MOIs, 0.1 or 0.001, and viral copies in the supernatant were detected. Upon infection, cells of both species showed cytopathic effects. Both cell lines showed high replication of VSV with maximum copy numbers in the range of 10^9^ viral copies per ml and lower maximum copy numbers for RVFV.

## Discussion

The present report describes the establishment of airway epithelial cell lines from two bat species pertaining to the suborders Yangochiroptera and Yinpterochiroptera for the study of bat-borne, including several zoonotic viruses. The detection of an increasing number of bat-borne viruses necessitates suitable model systems for the isolation and study of these viruses. Several new cell lines from bats have been established in recent years, but so far there has been no focus on targeted generation of epithelial cells [13], [14]. Successful cultures of primary and immortalized cells of airway epithelial origin are already well established for humans and a few animal species. These include mainly equine, porcine, bovine, and mouse cell lines [22]–[25], but to the best of our knowledge, no characterized airway epithelial cell lines from bats exist.

So far, only two bat cell lines are available from the American Type Culture Collection (ATCC): Tb 1 Lu, derived from the lung of the neotropical bat species *Tadarida brasiliensis* (Mexican free-tailed Bat), which was established in 1965; and Myi/It, derived from a skin tumor of the North American bat species *Myotis velifer incautus* (cave bat). However, given the high phylogenetic diversity of bats, these cell lines may not always be appropriate to study bat-related zoonotic viruses in detail. Cell lines are needed that allow the study of virus-host interaction in a wide range of host species; species that may differ in many aspects, including not only their geographic distribution, ecology, and biology, but also most likely in their immunological and receptor-associated characteristics as well.

Successful selective culturing of epithelial cells is dependent on the use of serum-free medium supplemented with hormones and growth factors [26]. Widely used formulations of cell culture medium supplemented with fetal bovine serum (FBS) are less suitable for epithelial cell growth, as, during the manufacturing process, serum platelet derived growth factor (PDGF) is released. In cell culture, PDGF has a stimulatory effect on fibroblasts, while it inhibits epithelial cell growth and quickly leads to terminal squamous differentiation in the primary cell culture [26]. Furthermore, even if epithelial cells are present in the primary cell culture, overgrowth of the epithelial proportion by fibroblasts after several passages can occur if medium containing FBS are used (unpublished observation). Most bat-derived cell lines published so far, however, were obtained and cultured using conventional medium containing FBS. It is therefore suggested that most of them contain at least a mix of several different cell types, including fibroblasts, or consist of fibroblasts only, as they normally overgrow other cell types after several passages. To prevent this, we used serum-free medium only for growth of primary cells, and found this approach to be efficacious. By contrast, Crameri et al. established primary and immortalized bat cell lines from *Pteropus alecto* and found serum-free medium the least successful approach in their culture compared to conventional medium formulations, however they do not comment on the cell type (i.e. epithelial vs. fibroblast) [13]. Their study primarily evaluated overall isolation success of primary cells but not targeted isolation of a certain cell type such as epithelial cells as presented here. Furthermore, there are various formulations of serum-free medium, which differ in their composition and are optimized for different primary cell types; it is therefore difficult to compare these findings.

In our approach, adaptation of a standard medium containing FBS was only performed after subcloning of immortalized cell cultures and repeated confirmation of the selected clones for epithelial cell markers. By using the method of endpoint limiting dilution, we ensured that each subclone originated from a single cell, so that contamination with fibroblasts or other non-epithelial cells could be excluded. Characterization of epithelial origin was additionally repeated in all subclones after adaptation to standard medium with FBS. Culture of the original immortalized cell lines was done with serum-free medium only until subcloning was performed.

In contrast to many cell lines obtained previously by us and other groups, we were able to culture and establish indefinite cell lines from an adult animal without the use of embryonic tissue. This has several advantages, as the use of embryonic tissue is more laborious and involves sampling of multiple individuals to obtain a gravid animal in the field. Further, separation of specific cell types is more difficult in embryos and contamination by multiple cell types is more likely.

Limitations of our method include the use of medium adapted to humans and human growth factors, which might not be optimal for other mammalian species such as bats. It is therefore likely that the cells may not retain all of the epithelial cell properties that are present *in vivo*. While it is unlikely that cell culture medium optimized for individual non-human species such as bats will ever become available, adaptation of protocols already successfully used for human and livestock cell cultures is a feasible approach. There is a general pitfall in all cell cultures, especially if further modifications such as immortalization or subcloning are performed. Thorough characterization of our cells showed that growth of the desired cell type was successful.

Recently, several findings supported the approach of isolation of bat-borne viruses in cell lines developed from the natural host species. Examples include the isolation of a henipa-related paramyxovirus in primary kidney cells from *Pteropus alecto* [27] as well as isolation of Menangle virus from the urine of the same species [28]. Zhang et al. showed isolation and growth of a bat herpesvirus from the bat *Miniopterus schreibersii* in primary bat cells after failure in 14 other mammalian cell lines [29]. Therefore bat cell lines could provide an opportunity for isolation and characterization of bat-borne viruses that otherwise fail to grow in mammalian cell lines.

On the other hand, bat cell lines can provide a hint for zoonotic origin of human viruses, for example Huynh and coworkers showed replication of HCoV-NL63 in immortalized lung cells from the North American tri-colored bat (*Perimyotis subflavus*) for multiple passages and therefore conclude a bat origin of the virus [30].

Due to the large number of species that build the order Chiroptera, it will not be possible to find a perfect match between virus and natural host in all instances – however; there are examples of virus receptors that are conserved between different members of the Chiropteran order, and therefore bat cell lines that are representatives of the order can serve as valuable representatives. The most recent example is the conserved receptor Dipeptidyl-peptidase-4 for MERS-coronavirus that allows infection of bat cell lines, not only originating from the presumed reservoir host, bats of the family Vespertilionidae, but across several other bat families [31].

To study airborne transmission of viruses in a system that resembles the *in vivo* situation as closely as possible, we aimed at establishing a protocol for generating primary cell lines as well as indefinite cell cultures. Even though generation of primary cells from repeated outgrowth of trachea specimens was successful, only a small number of cells were obtained in total from a single animal, limiting complex experiments with primary cells. This problem could be overcome by culturing organ specimens from multiple animals; but for almost all relevant wild zoonotic species it is not possible to harvest material of the required quality from a large number of individuals. The generation of immortalized cell lines that are not limited in their passage number can overcome this problem but the immortalization process comes at the cost of interfering with at least some primary cell properties. As shown, immortalized cell lines and cell lines from a subcloned single cell still maintain many of their original characteristics, such as confluent growth as a monolayer and expression of tight junction proteins, an important feature of epithelial cells. Furthermore, immortalized cell lines are valuable and easy to maintain. They can therefore be shared with other groups, facilitating zoonotic research beyond the lab of origin. Finally, adapting single cell clones of confirmed epithelial origin to standard medium will allow for less costly propagation and use compared with cells adapted to serum-free primary cell medium.

With these issues in mind, we believe the protocols and cell cultures developed in the present study will provide a useful model for the study of bat-borne viruses and virus-host interaction in bats.

## Supporting Information

Table S1 **Virus families and genera for which screening by nucleic acid amplification was performed. Details on assays available upon request.**
(DOCX)

Video S1 **Movement of ciliae in primary cell culture (here: *E. helvum*).**
(AVI)
