# Supplementary material for: Bat Airway Epithelial Cells: A Novel Tool for the Study of Zoonotic Viruses
Source: PLoS One. 2014 Jan 13;9(1):e84679. doi: 10.1371/journal.pone.0084679 (PMC3890267; doi:10.1371/journal.pone.0084679)
Supplement: Table S1 — Virus families and genera for which screening by nucleic acid amplification was performed. Details on assays available upon request. (DOCX) [file pone.0084679.s001.docx]

**Table 1S.** Virus families and genera for which screening by nucleic acid amplification was performed. Details on assays available upon request.

| **RNA viruses** |
| --- |
| ***+ strand RNA*** |
| Genus *Cardiovirus* |
| Genus *Enterovirus* |
| Genus *Parechovirus* |
| Genus *Flavivirus* |
| Genus *Pestivirus* |
| Genus *Norovirus* |
| Genus *Astrovirus* |
| Genus *Hepevirus* |
| Genus *Coronavirus* |
| Genus *Alphavirus* |
| ***- strand RNA*** |
| Family *Filoviridae* |
| Family *Paramyxoviridae* |
| Genus *Influenzavirus A, B, C* |
| Genus *Nairovirus* |
| Genus *Phlebovirus* |
| Genus *Orthobunyavirus* |
| Genus *Lyssavirus* |
| Genus *Bornavirus* |
| Genus *Hantavirus* |
| ***ds RNA*** |
| Genus *Lentivirus* |
| Genus *Rotavirus* |
| Genus *Orbivirus* |
| Genus *Orthoreovirus* |
| ***+/- strand RNA*** |
| Family *Arenaviridae* |
| ***DNA viruses*** |
| Familiy *Hepadnavirus* |
| Genus *Alpha-Herpesvirus* |
| Genus *Adenovirus* |
| Subfamily *Chordopoxvirus* |
